# Meta-analysis reveals that seed-applied neonicotinoids and pyrethroids have similar negative effects on abundance of arthropod natural enemies

**DOI:** 10.7717/peerj.2776

**Published:** 2016-12-07

**Authors:** Margaret R. Douglas, John F. Tooker

**Affiliations:** Department of Entomology, The Pennsylvania State University, University Park, PA, United States

**Keywords:** Meta-analysis, Biological control, Neonicotinoids, Pyrethroids, Natural enemies, Predator, Parasitoid, Ecosystem services, Systemic insecticides, Field crops

## Abstract

**Background:**

Seed-applied neonicotinoids are widely used in agriculture, yet their effects on non-target species remain incompletely understood. One important group of non-target species is arthropod natural enemies (predators and parasitoids), which contribute considerably to suppression of crop pests. We hypothesized that seed-applied neonicotinoids reduce natural-enemy abundance, but not as strongly as alternative insecticide options such as soil- and foliar-applied pyrethroids. Furthermore we hypothesized that seed-applied neonicotinoids affect natural enemies through a combination of toxin exposure and prey scarcity.

**Methods:**

To test our hypotheses, we compiled datasets comprising observations from randomized field studies in North America and Europe that compared natural-enemy abundance in plots that were planted with seed-applied neonicotinoids to control plots that were either (1) managed without insecticides (20 studies, 56 site-years, 607 observations) or (2) managed with pyrethroid insecticides (eight studies, 15 site-years, 384 observations). Using the effect size Hedge’s *d* as the response variable, we used meta-regression to estimate the overall effect of seed-applied neonicotinoids on natural-enemy abundance and to test the influence of potential moderating factors.

**Results:**

Seed-applied neonicotinoids reduced the abundance of arthropod natural enemies compared to untreated controls (*d* = −0.30 ± 0.10 [95% confidence interval]), and as predicted under toxin exposure this effect was stronger for insect than for non-insect taxa (*Q_M_* = 8.70, df = 1, *P* = 0.003). Moreover, seed-applied neonicotinoids affected the abundance of arthropod natural enemies similarly to soil- or foliar-applied pyrethroids (*d* = 0.16 ± 0.42 or −0.02 ± 0.12; with or without one outlying study). Effect sizes were surprisingly consistent across both datasets (*I*_2_ = 2.7% for no-insecticide controls; *I*_2_ = 0% for pyrethroid controls), suggesting little moderating influence of crop species, neonicotinoid active ingredients, or methodological choices.

**Discussion:**

Our meta-analysis of nearly 1,000 observations from North American and European field studies revealed that seed-applied neonicotinoids reduced the abundance of arthropod natural enemies similarly to broadcast applications of pyrethroid insecticides. These findings suggest that substituting pyrethroids for seed-applied neonicotinoids, or vice versa, will have little net affect on natural enemy abundance. Consistent with previous lab work, our results also suggest that seed-applied neonicotinoids are less toxic to spiders and mites, which can contribute substantially to biological control in many agricultural systems. Finally, our ability to interpret the negative effect of neonicotinoids on natural enemies is constrained by difficulty relating natural-enemy abundance to biological control function; this is an important area for future study.

## Introduction

Arthropod natural enemies (predators and parasitoids) contribute considerable value to agriculture by suppressing pests that attack crop plants. For example, biological control of the soybean aphid (*Aphis glycines*) is estimated to be worth at least $84 million per year in just five US states (Zhang & Swinton, 2012). Given their importance to pest management, it is essential to understand how agricultural practices influence natural-enemy communities and their ability to suppress crop pests. Insecticide use is one common agricultural practice that can influence natural-enemy populations and biological control. Insecticides are used to manage pests, however, in some cases they also disrupt biological control, leading to unintended outbreaks of target or non-target pests (Geiger et al., 2010; Settle et al., 1996; Stern et al., 1959). Elucidating how insecticides and natural enemies interact can be useful in designing stable pest management strategies, identifying insecticide products that have greater selectivity for pests versus natural enemies, applying them in ways that conserve natural enemies, and in some cases even using insecticides to enhance the efficacy of biological control (Croft & Brown, 1975; Hull & Beers, 1985).

One use of insecticides that is increasingly widespread is planting seeds coated with neonicotinoid insecticides, especially in large-acreage field crops where these seed coatings are ubiquitous in some crops and regions (Simon-Delso et al., 2015). Seed coatings account for at least 80% of neonicotinoids applied in the US (Douglas & Tooker, 2015) and accounted for roughly 87% in Britain prior to recent restrictions (Simon-Delso et al., 2015). Through their systemic activity, seed-applied neonicotinoids target some early-season soil and foliar pests, and their relatively low cost and ease of handling makes them an attractive option as ‘insurance’ against sporadic pest populations (Douglas & Tooker, 2015; Jeschke et al., 2011). The popularity of neonicotinoids is also related to their toxicological profile; their binding behavior at nicotinic acetylcholine receptors confers potent toxicity against a broad array of insect species, and simultaneously low acute toxicity to mammals (Tomizawa & Casida, 2005).

Despite the broad toxicity of neonicotinoids to insects, some researchers reasoned that by applying them in relatively small doses ‘targeted’ to crop seed, seed-applied neonicotinoids would have high ecological selectivity for crop pests and low potential to reduce populations of beneficial insects (Jeschke et al., 2011). The environmental effects of these products have since been called into question by evidence that non-target species, especially bees, can be exposed to seed-applied neonicotinoids via contaminated soil, planting dust, floral resources, and guttation droplets (Godfray et al., 2014; Godfray et al., 2015). This pollinator controversy has spurred new regulations in several jurisdictions (European Commission, 2013; Government of Ontario, 2015), in turn raising the question of whether other pest management tactics that might replace neonicotinoids, including conventionally applied insecticides, have more or less influence on non-target species. We argue that fully understanding the trade-offs associated with seed-applied neonicotinoids requires attention not only to pollinators but also to natural enemies that suppress crop pests.

Seed-applied neonicotinoids can potentially reduce populations of arthropod natural enemies through at least two mechanisms: toxin exposure and prey scarcity. Neonicotinoids are toxic to many natural-enemy species (Cloyd & Bethke, 2011; Hopwood et al., 2013), but it is unclear whether under field conditions natural enemies encounter meaningful doses of toxins from seed treatments. What constitutes a ‘meaningful dose’ is likely to vary by taxon. In the laboratory, insects are orders of magnitude more susceptible to neonicotinoids than arachnids (Table 1), and at least some evidence suggests that this difference is based on the molecular structure of arachnid acetylcholine receptors (Meng et al., 2015). If natural-enemy populations were reduced by seed-applied neonicotinoids through toxin exposure, we would expect insects to be more strongly affected than arachnids. Alternatively, neonicotinoids could exert indirect negative effects, for instance by reducing the abundance of prey, leading to less aggregation, persistence, or reproduction of natural enemies in crop fields (Croft & Brown, 1975). Under this prey-scarcity scenario, we would expect insects and arachnids to be similarly affected, but functional groups to differ relative to their degree of dependence on pest prey (parasitoid > predator > omnivore). These two types of effects of insecticides on natural enemies are not mutually exclusive, and both can interfere with biological control (Johnson & Tabashnik, 1999). Clarifying the mechanisms at play can nonetheless guide researchers and pest managers toward more successful integration of chemical and biological control.

**Table 1:** LC_50_ results from two laboratory studies that compared imidacloprid toxicity to insect and arachnid predators.

Study	Class	Order	Family	Species	Life stage	LC_50_ (ppm)
Mizell & Sconyers (1992)^a^	Arachnida	Mesostigmata	Phytoseiidae	*Neoseiulus collegae*	Adult females	>12,744
				*Phytoseiulus macropilus*	Adult females	3,561
				*Proprioseiopsis mexacanus*	Adult females	>1,274
	Insecta	Coleoptera	Coccinellidae	*Olla v-nigrum*	Adults	3.07
					Last instar larva	2.62
		Hemiptera	Miridae	*Deraeocoris nebulosus*	Adults	0.0163
		Neuroptera	Chrysopidae	*Chrysoperla rufilabris*	Adults (pop 1)	190
					Adults (pop 2)	155
					Eggs	20.2
Tanaka, Endo & Kazano (2000)^b^	Arachnida	Araneae	Linyphiidae	*Gnathonarium exsiccatum*	1st instar nymphs	801
				*Ummeliata insecticeps*	1st instar nymphs	995
			Lycosidae	*Pardosa pseudoannulata*	1st instar nymphs	440
			Tetragnathidae	*Tetragnatha maxillosa*	1st instar nymphs	136
	Insecta	Hemiptera	Miridae	*Cyrtorhinus lividipennis*	Adult females	0.36
		Hymenoptera	Dryinidae	*Haplogonatopus apicalis*	Adult females	0.12

**Notes.**

a Residual toxicity; predators were exposed to imidacloprid residues on petri dishes for 48–72 h.

b Contact toxicity; predators were immersed in insecticide solution and mortality was measured after 24–48 h.

Field experiments on the influence of seed-applied neonicotinoids on natural enemies have reached mixed conclusions. Some have found no statistically significant effects (Al-Deeb et al., 2003), some a mix of null and negative effects (Albajes, Lopez & Pons, 2003), and others more consistent negative effects (Hallett et al., 2014). Some of this variability may be related to the small sample sizes of most field studies, which limits their statistical power. Conversely, variability in study results may reflect real differences across crop species, natural-enemy taxa, active ingredients, and other factors. One powerful tool to make sense of such apparently mixed results and to untangle the various factors influencing study outcomes is meta-analysis (Cumming, 2012). This approach has been fruitfully applied in similar situations, for instance, to estimate the influence of *Bt* transgenic crops on non-target organisms (Marvier et al., 2007; Naranjo, 2009; Wolfenbarger et al., 2008). One of the salient benefits of meta-analysis in controversial areas is that it provides a rigorous, quantitative, and replicable method of synthesizing evidence for researchers, policy-makers, and the public (Marvier et al., 2007).

Here, we report results from a meta-analysis of studies investigating under field conditions the influence of seed-applied neonicotinoids on arthropod natural enemies. We used a meta-regression approach to test the hypotheses that seed-applied neonicotinoids: (1) negatively affect natural-enemy abundance relative to untreated controls; (2) reduce natural enemy abundance less than conventional foliar- or soil-applied insecticide treatments; and (3) affect natural enemies through a combination of toxin exposure and prey scarcity. Our results taken together should allow researchers and pest managers to better predict the compatibility of seed-applied neonicotinoids and natural enemies, and to more effectively weigh the use of these insecticides against alternative pest-management options.

## Materials & Methods

Meta-analysis is a method for synthesizing observations from independent yet similar studies to characterize the size and variability of an effect—in this case the influence of seed-applied neonicotinoids on natural enemies of crop pests. Our meta-analysis was guided to some extent by previous meta-analyses that characterized the influence of *Bt* crops on populations of non-target arthropods (Marvier et al., 2007; Naranjo, 2009; Wolfenbarger et al., 2008). We departed from these previous studies in our statistical approach. In particular, as described below, we capitalized on advances in statistical programs over the past several years to better account for the hierarchical nature of the dataset. All of our analyses were conducted within the R statistical program (R Core Team, 2015).

### Analysis procedure

#### Searching the literature

Using four databases (ISI Web of Science, Agricola, CAB abstracts, and ProQuest Dissertations & Theses Database), we searched for studies on the influence of neonicotinoids on arthropod natural enemies. We used the following search phrase, adjusting the syntax as necessary for different search engines: “(neonic* OR imidacloprid OR clothianidin OR thiamethoxam) AND (preda* OR enem* OR parasit*) AND seed”. We combed the resulting studies and published reviews (Chagnon et al., 2015; Hopwood et al., 2013; Lundgren, 2009; Pisa et al., 2015) for additional references, and found one additional unpublished thesis because a colleague mentioned it at a scientific meeting. Our final literature search for this analysis was conducted on August 7, 2015. The search was designed by both authors and carried out by the first author.

#### Building the dataset

We used the following criteria to include a study in the dataset: (1) it compared field plots that were planted with neonicotinoid-treated seed with control plots that were planted with neonicotinoid-free seed of the same crop variety. There were two types of control plots: those that were not treated with any insecticides (testing whether neonicotinoids have any effect on natural enemies), and those that were treated with an alternative insecticide product (testing whether neonicotinoids affect natural enemies more or less than alternatives). Studies also had to (2) measure abundance or activity-density of one or more taxonomic groups of arthropod natural enemy, (3) be replicated, (4) report the data necessary to calculate effect size (means, sample sizes, and standard errors or standard deviations), and (5) be available in English. Where studies met the first three criteria but did not report some necessary data, we contacted authors to obtain that data, although not all responded. Where necessary we extracted data from published figures using the software GraphClick 3.0.3 (Arizona Software).

To build the dataset, we recorded for each study the means and variability for natural-enemy abundance in each treatment group, along with a wide variety of supporting information such as author and affiliation, study location and year, crop species, active ingredient of the seed treatment, size of each plot, number of replicates, and other methodological details. While seed-applied neonicotinoids should have their largest effects during the early growing season, we found that studies varied in their emphasis on this early sampling window, and some studies did not even start sampling until mid-season (Table S1). If a taxon was sampled repeatedly over a single season, we used seasonal summary data when available; otherwise we requested seasonal data from the authors. If this failed, we used peak values. We chose to use seasonal and peak values to be consistent with previous meta-analyses (e.g., Marvier et al., 2007), and to ensure a consistent basis for comparing seed-applied neonicotinoids to pyrethroid applications, which sometimes occur later in the season. Nonetheless, we tested for the potential influence of sampling window (% of sample from the early season) in our statistical analysis, as described below in “Fitting meta-regression models.”

For each taxon, we recorded sampling method, life stage, habitat (soil/epigeal or foliar/aerial), functional group, and taxonomic information to the lowest level provided. Many natural enemies consume plant products (pollen, nectar, seeds, etc.) in addition to live prey; we assigned taxa to functional groups using an existing classification (Wolfenbarger et al., 2008), and filling in gaps where necessary based on the scientific literature. We defined ‘natural enemies’ to include the following functional groups of Wolfenbarger et al. (2008): mixed, omnivore, predator, and parasitoid. The ‘omnivore’ group comprises taxa that are believed to rely equally on prey and non-prey foods (e.g., Formicidae, Gryllidae). The ‘mixed’ group refers to taxonomic units that contain species in multiple functional groups (e.g., Carabidae). We classified natural enemies into functional groups based on the taxonomic level at which the data were reported, which varied from class to species (see Data S1 for a full list of functional group classifications).

#### Defining the scope of the study

While our initial intent was to include studies from all geographic regions, we restricted the current analysis to North America and Europe because most of the studies from other regions (eight of 11 studies, all from South Asia) lacked sufficient details for us to interpret reported measures of variation. For the part of our analysis comparing neonicotinoids to alternative insecticides, we restricted the analysis to pyrethroids, the only insecticide class that was compared to seed-applied neonicotinoids in at least three independent studies (Table S1). Because seed-application is far more common for neonicotinoids than other classes of insecticides (Douglas & Tooker, 2015), we expected pyrethroid comparisons to be applied using traditional broadcast methods such as foliar sprays or granular soil applications. This was mostly true, but there was one study that applied pyrethroids as a seed treatment (Baker et al., 2002). For completeness, we included this study in our analyses as a ‘soil-based’ pyrethroid, but also tested the sensitivity of our results to its inclusion.

#### Calculating effect size: Hedge’s d

The response variable in our analysis was Hedge’s *d*, the mean abundance in the control group minus the mean abundance in the treatment group, divided by the pooled standard deviation, and corrected for small sample size (Koricheva, Gurevitch & Mengersen, 2013). In this case *d* measured the difference in natural-enemy abundance between control plots and plots planted with neonicotinoid-treated seed, with negative values reflecting lower abundance of natural enemies in neonicotinoid-treated plots compared to controls. We used the ‘escalc’ function (‘metafor’ package) to calculate *d* and its associated variance (*σ*^2^) for each observation in the dataset.

#### Addressing non-independence

Typical studies in our dataset contributed numerous observations resulting from multiple taxa, sampling methods, life stages, site-years, and other factors. As a result we could not assume that all of the observations were statistically independent. As in past meta-analyses (Marvier et al., 2007), we addressed this problem in part by eliminating redundant observations, as follows. When results were reported at varying levels of taxonomic resolution, we used only the results at the finest taxonomic level. When multiple life stages were sampled for a given taxon, we used only the observations from the least mobile, but still feeding life stage (Wolfenbarger et al., 2008). When a given taxon was sampled in multiple ways, we included results from the sampling method with the highest precision (lowest relative standard deviation). We made an exception to this rule for studies that sampled soil and foliar habitats for Araneae, “predatory mites” and Carabidae, because for these broad taxonomic groups, these habitats are likely to contain mostly non-overlapping taxa. Even after taking these steps to reduce redundancy, our dataset still contained numerous observations per study as a result of multiple taxa, site-years, and crossed factors such as crop varieties and insecticide active ingredients. We accounted for the remaining non-independence through the structure of the meta-regression models as described in the next section.

#### Fitting meta-regression models

To estimate the influence of seed-applied neonicotinoids on natural enemies and to test the influence of agro-ecological or methodological moderating variables on the size of this effect, we employed a mixed effects meta-regression approach using the package ‘metafor’ in R (R Core Team, 2015; Viechtbauer, 2010). Meta-regression is analogous to multiple regression, but differs in that observations are weighted relative to their precision (typically 1/variance). The strength of the meta-regression approach is that it allows us to investigate the influence of multiple moderators at once, while also using random effects to control for the hierarchical nature of the dataset (observations nested in site-years nested in studies).

We split our larger dataset into two parts: one for observations that compared seed-applied neonicotinoids to an insecticide-free control and a second for observations that compared seed-applied neonicotinoids to pyrethroids (Data S1). For each of these datasets we fit three models, all estimated using restricted maximum likelihood in ‘metafor’ (Codes S1 and S2). First we fit a ‘null’ model that did not include random effects of site-year or study, nor fixed effects of moderators. This model mainly serves as comparison to results of previous meta-analyses on non-target effects of agricultural technologies, many of which did not account for nesting of observations within studies. We next fit a ‘site-year/study’ model that included only random effects of site-year and study, but no fixed effects (i.e., no moderators). From these two initial sets of models we generated 95% confidence intervals for the overall influence of seed-applied neonicotinoids on natural enemies, and also characterized variability in the effect sizes using ‘heterogeneity’ as measured by the *Q* statistic. *Q* is the weighted sum of squared differences of effect sizes from the mean, and can be used to test whether variability among effect sizes is greater than would be expected by sampling error alone (Viechtbauer, 2007b). In addition to the *Q* test, we calculated *I*^2^ = 100% × (*Q* − df)∕*Q*, where df = degrees of freedom (*k* − 1; *k* = the total number of observations), which estimates the percentage of variability in effect sizes that is due to true heterogeneity rather than sampling error (Higgins et al., 2003). Finally, to assess whether it was necessary to include the study and site-year effects, we examined the variance components associated with these effects in the ‘site-year/study’ model using profile plots.

The third model we fit for each dataset was a ‘moderator’ model designed to test whether agro-ecological or methodological variables influenced the effect of seed-applied neonicotinoids on natural enemies. Along with random effects of site-year and study, these models included fixed effects of potential moderating variables that we identified *a priori* (See Table 2 for details): broad taxonomic group (insect or non-insect arthropod), functional group (predator, parasitoid, omnivore, or mixed), habitat (soil-associated or foliage-associated), neonicotinoid active ingredient group (imidacloprid or clothianidin/thiamethoxam), crop species (maize, soybean, or other), publication status (peer-reviewed or dissertation/thesis/other), pyrethroid application method (where applicable; soil or foliar application), plot size, and proportion of samples collected during the first 40 days of crop growth, when neonicotinoids typically have activity against target pests (Magalhaes, Hunt & Siegfried, 2009; Seagraves & Lundgren, 2012). For the pyrethroid analysis, to reduce the risk of drawing spurious conclusions we left out crop species, publication status, and functional group, because some levels of these moderators were not supported by at least three independent studies (Table 2). We transformed continuous moderators (plot size, proportion early samples) where necessary and centered them on a mean of zero to facilitate interpretation. Categorical variables were converted to effects coding by employing the ‘contr.sum’ option in ‘contrasts.’ This made the intercept of the fitted model reflect the mean value across the means of all moderator variables, and the slopes reflect the difference associated with the level of each moderator from the overall mean. We first tested for the significance of all the moderators combined (using an omnibus *Q* test for moderators); when that was significant, we went on to test the significance of individual moderators.

**Table 2:** Description of the dataset used in a meta-analysis of seed-applied neonicotinoid effects on natural enemies of crop pests.

		No insecticide control			Pyrethroid control		
Variable	Levels	Studies	Site-years	Obs. (%)^a^	Studies	Site-years	Obs. (%)^a^
Taxonomic group	Insects	20	56	493 (81%)	8	15	313 (82%)
	Non-insect arthropods	14	30	114 (19%)	6	11	71 (18%)
Habitat	Soil-associated	11	26	189 (31%)	6	10	156 (41%)
	Aboveground	15	48	418 (69%)	5	11	228 (59%)
Functional group	Omnivore	6	13	39 (6%)	4	8	41 (11%)
	Mixed	12	32	79 (13%)	5	8	46 (12%)
	Predator	17	48	408 (67%)	8	15	262 (68%)
	Parasitoid	7	27	81 (13%)	2	6	35 (9%)
Active ingredient	Imidacloprid	11	29	336 (55%)	6	12	279 (73%)
	Clothianidin/Thiamethoxam	13	35	271 (45%)	6	10	105 (27%)
Crop species	Corn	7	20	300 (49%)	4	10	244 (64%)
	Soybeans	7	22	200 (33%)	2	5	114 (30%)
	Other	6	14	107 (18%)	2	3	26 (7%)
Publication status	Peer-reviewed journal	13	36	459 (76%)	6	12	358 (93%)
	Diss./Thesis/Other	7	20	148 (24%)	2	3	26 (7%)
Pyrethroid application	Soil-based	–	–	–	5	8	159 (41%)
	Foliar	–	–	–	4	10	225 (59%)
TOTAL		20	56	607 (100%)	8	15	384 (100%)

**Notes.**

a Number of observations in each category, followed by the percentage of values in the dataset in that category.

We tested the moderators that we expected to have the greatest likelihood of influencing effect size, while limiting the total number of moderators to preserve the power of the analysis. As described in the introduction, effects of taxonomic group and functional group have implications for whether natural enemies are affected by neonicotinoids through toxin exposure (insects > arachnids) and/or prey scarcity (parasitoid > predator > omnivore). Habitat, active ingredient, and crop species may mediate the influence of neonicotinoids on natural enemies because these factors likely correspond to differences in exposure, toxicity, and prey communities. We included publication status because a relationship between publication status and effect size could indicate publication bias (Koricheva, Gurevitch & Mengersen, 2013). Finally, we included plot size, early-season sampling, and pyrethroid application method in the model to control for methodological variables that we suspected might influence research outcomes.

#### Assessing statistical assumptions, potential biases, and robustness of results

As in multiple regression, in meta-regression correlations among moderator variables (collinearity) can render estimates and tests of model parameters unreliable (Kutner et al., 2005; Viechtbauer, 2007a). Before fitting the ‘moderator’ models, we first calculated and examined pairwise correlations among all our moderators. We also examined generalized variance inflation factors (GVIF) by fitting linear models using the ‘lme’ function (nlme package) with our moderators in the models as fixed effects and site-year nested in study as random effects. We used the ‘vif’ function (car package) to calculate GVIF values.

To further assess the fit of our models, we examined the standardized residuals versus fits and inspected the normality of the residuals using a QQ plot. To screen for influential observations, we plotted leverage values on their own and relative to residuals. When we found potential outliers, we re-fit our models without them to assess their influence on our conclusions.

We tested for publication bias in part through the ‘publication status’ moderator in the meta-regressions, as discussed above. Additionally we examined a weighted histogram of the effect sizes for evidence of ‘missing’ observations near zero, and used the ‘trimfill’ and ‘funnel’ functions (‘metafor’ package) on our null models to generate funnel plots and test for ‘missing’ observations in the distributions. Finally to test the robustness of the overall effect size estimates, when they differed from zero, we calculated Rosenberg’s ‘fail safe N’, that is, the number of null observations necessary to make the observed effect size non-significant. We note that most of these methods for testing publication bias do not take into account the hierarchical nature of our dataset; there do not appear to be tools available that explicitly account for this data structure.

To test the sensitivity of our results to inclusion of particular studies, we conducted a ‘leave one out’ analysis in which we removed the observations associated with each study from each dataset one by one, and then re-fit all three models. We assessed the consistency of the confidence intervals for the overall effects, as well as the fitted slopes and hypothesis tests for the ‘moderator’ models. Where eliminating a study changed the interpretation of our analysis, we noted this in the results.

#### Predator-prey ratios

Because insecticides can affect pest and predator populations differently, predator–prey ratios may be more reflective of biological control function than predator abundance alone (e.g., Croft & Nelson, 1972; Naranjo, 2005; Ooi, 1982). Unfortunately most studies did not report predator–prey ratios or sufficient data to calculate them, but we were able to perform a preliminary summary based on soybean studies, which more often reported cumulative abundance of both pests and relevant predators. For the soybean studies that reported both the cumulative abundance of a pest taxon and the cumulative abundance of the predator guild for that pest, we calculated predator–prey ratios for neonicotinoid treatments and controls. Without access to the original data it was impossible to estimate a variance for the predator–prey ratios, so we did not perform a formal meta-analysis; instead we discuss them qualitatively to lend preliminary insight into the relative impact of neonicotinoids on predator and pest populations. To facilitate this summary, we calculated the percent change in the predator–prey ratio in each neonicotinoid treatment relative to each control. Negative values indicate that seed-applied neonicotinoids reduced the predator–prey ratio relative to the control, while positive values indicate the opposite.

## Results

### Results of the literature search & characteristics of the meta-analysis dataset

**Figure 1:**
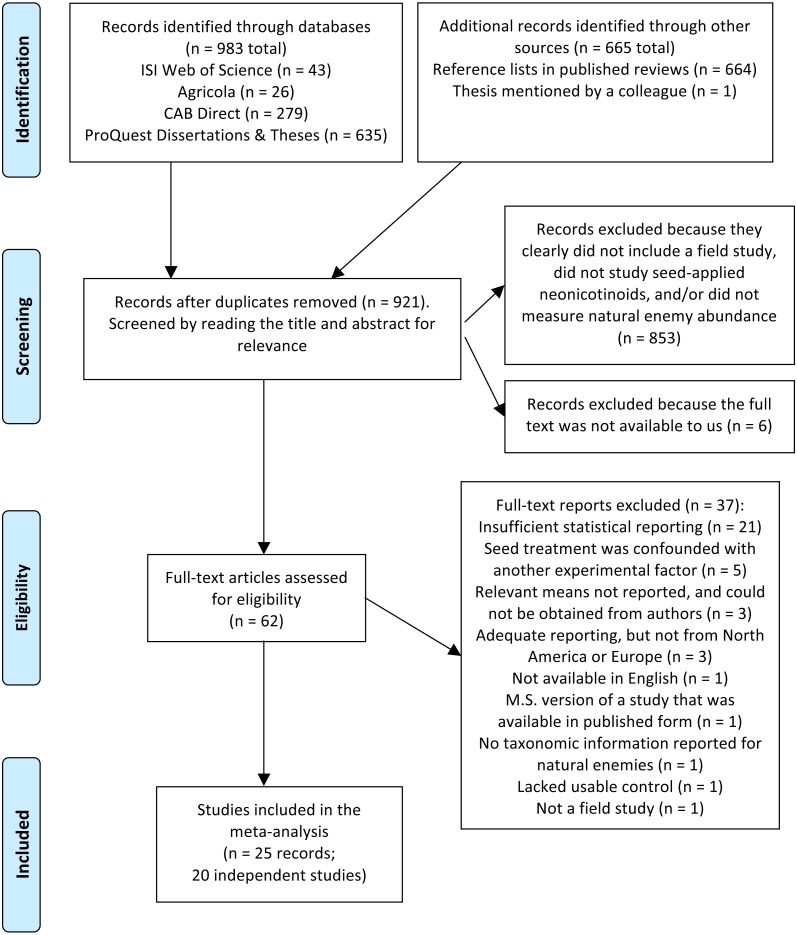
PRISMA diagram. Flow diagram based on the preferred reporting items for systemic reviews and meta-analyses (PRISMA; Moher et al., 2009), describing the literature search and screen used to identify studies for a meta-analysis on the influence of seed-applied neonicotinoids on natural enemies.

In total we screened 921 titles and abstracts, yielding 62 candidate reports (Fig. 1). After assessing eligibility, filtering for relevant functional groups and geographic regions (North America and Europe), and reducing redundant observations, our final dataset for the no-insecticide controls comprised 607 observations collected over 56 site-years and 20 independent studies (Table S1 and References S1). For the pyrethroid controls, our final dataset comprised 384 observations collected over 15 site-years and 8 independent studies (Table S1 and References S1). Corn (*Zea mays*) and soybean (*Glycine max*) were the dominant crops, and insects were better represented in the dataset than non-insect arthropods (arachnids and chilopods; Table 2). Unsurprisingly, given the focus of our study, predators were the dominant functional group (67–68% of observations), although parasitoids, omnivores, and mixed functional groups were also represented. Observations were spread fairly equally among soil and foliar habitats, different active ingredients, and pyrethroid application methods, and most observations were derived from peer-reviewed studies (76–93%; Table 2). Plot size ranged widely from 1 to 110,000 m^2^, and proportion of early season sampling ranged from zero to 100% (Table S1). Most studies had a sample size of three to six replicate plots per treatment (Table S1).

### Seed-applied neonicotinoids negatively affected natural enemies compared to no-insecticide controls

**Figure 2:**
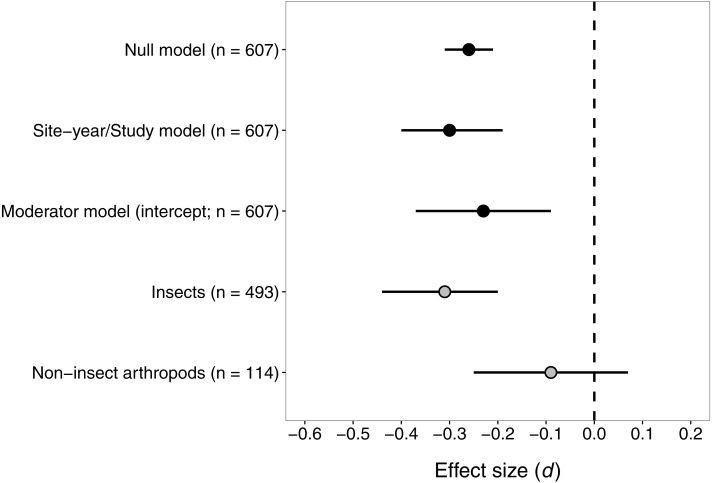
Confidence intervals (95%) for the effect of seed-applied neonicotinoids on natural-enemy abundance, relative to no-insecticide controls. *n*, the number of observations associated with each estimate; observations were derived from 56 site-years and 20 studies (see Table 2 for further description of the dataset). See text for details on models used to generate these estimates.

Consistent with our hypothesis, seed-applied neonicotinoids reduced abundance of arthropod natural enemies relative to untreated controls (Fig. 2). The mean effect size (*d*) was −0.30 or −0.26, for the ‘site-year/study’ and ‘null’ models respectively. For context, an effect size of −0.30 would correspond to an approximate reduction of 16% in natural-enemy abundance (−0.30 ×median relative standard deviation [RSD] of 0.53). The estimates of the variance components from the fitted model suggested that site-year explained most of the shared variation among observations within studies (study *σ*^2^ = 0.0035, site-year *σ*^2^ = 0.08). The negative effect of seed-applied neonicotinoids on natural enemies appeared to be homogenous (*Q* = 622.5, df = 606, *P* = 0.31), with an *I*^2^ indicating that all but 2.7% of variation in effect sizes could be explained by random sampling error.

Despite the low heterogeneity identified in the initial analysis, we proceeded with the ‘moderator’ analysis to test the influence of various factors on the effect of seed-applied neonicotinoids on natural enemies. We did this because we had planned the moderator analysis *a priori*, and because the *Q* test, despite being the most powerful test available, still has low power to detect heterogeneity in datasets like ours where the within-study sample sizes are small (Viechtbauer, 2007b). Indeed, when we fit the meta-regression model with our eight moderator variables, the omnibus test suggested that the moderators taken together did explain significant variation in effect size (*Q*_M_ = 21.5, df = 11, *P* = 0.029). Broad taxonomic group apparently drove this result, as it was the only moderator that was significant when tested individually (Table 3). As predicted under toxin exposure, the negative effect of seed-applied neonicotinoids on natural enemies was stronger for insects than for non-insect taxa (mostly spiders and mites; Fig. 2). When estimated separately, the negative effect of neonicotinoids on insects remained significant, while the effect on non-insect taxa did not differ significantly from zero (Fig. 2). Although functional group did not significantly moderate the influence of seed-applied neonicotinoids on natural enemies (*P* = 0.13), the fitted slopes for this moderator were fairly large and followed a trend consistent with indirect effects via prey scarcity (parasitoid > predator > omnivore, Table 3). Surprisingly, effect size was not influenced to a significant extent by crop species, neonicotinoid active ingredient, habitat, or any of the methodological variables (Table 3).

**Table 3:** Estimates and tests of significance for moderators in a meta-regression model testing the effect of seed-applied neonicotinoids on natural enemies, compared to controls treated with no insecticides (*n* = 607 observations from 56 site-years and 20 studies).

Moderator	Level	*β*	*Q*_M_	df	*P* value
Intercept	–	−0.23	–	–	–
Taxonomic group	–		8.70	1	**0.003**
	Insects	−0.11			
	Non-insect arthropods	0.11			
Habitat	–		1.42	1	0.23
	Aboveground	0.057			
	Soil-associated	−0.057			
Functional group	–		5.61	3	0.13
	Omnivore	0.186			
	Mixed	0.049			
	Predator	−0.071			
	Parasitoid	−0.164			
Crop species	–		0.79	2	0.67
	Corn (*Zea mays*)	0.072			
	Soybean (*Glycine max*)	0.002			
	Other	−0.074			
Active ingredient	–		0.99	1	0.32
	Imidacloprid	0.043			
	Clothianidin/Thiamethoxam	−0.043			
Publication type	–		0.51	1	0.56
	Peer-review journal	0.062			
	Dissertation/Thesis/Other	−0.062			
ln(Plot size)	–	−0.016	0.34	1	0.56
ln(Early sampling + 0.1)	–	−0.076	0.61	1	0.44

### Seed-applied neonicotinoids reduced natural enemy abundance similarly to pyrethroid insecticides

**Figure 3:**
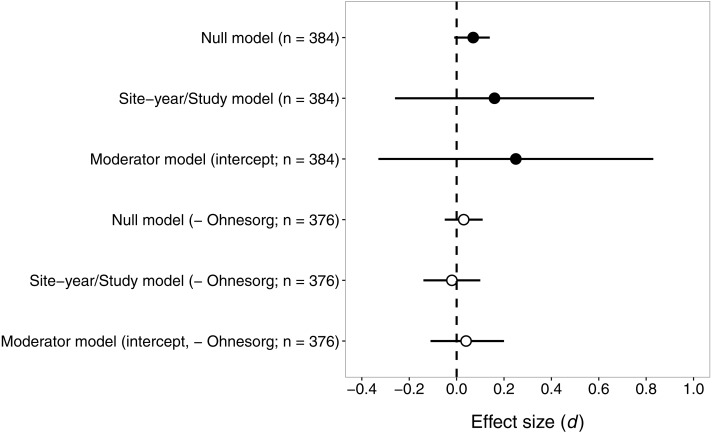
Confidence intervals (95%) for the effect of seed-applied neonicotinoids on natural-enemy abundance, relative to controls treated with foliar or soil-applied pyrethroids. *n*, the number of observations associated with each estimate; observations were derived from 15 site-years and eight studies (see Table 2 for further description of the dataset). Results are presented both with and without data from (Ohnesorg, Johnson & O’Neal, 2009), which had effect sizes quite different from the other studies. See text for details on models used to generate these estimates.

Contrary to our prediction, the effect size for pyrethroids versus seed-applied neonicotinoids did not differ significantly from zero (Fig. 3), suggesting that these two groups of insecticides reduce natural-enemy abundance to a similar extent. The sensitivity analysis revealed that one study (Ohnesorg, Johnson & O’Neal, 2009) had a large influence on effect sizes and confidence intervals, so we present results both with and without this study (Fig. 3).

With all studies in the dataset, the mean effect size (*d*) was 0.16 or 0.07, for models that did or did not include random effects of site-year nested in study (Fig. 3). Including random effects for site-year and study made the confidence intervals very wide, because of the influence of Ohnesorg, Johnson & O’Neal (2009). Similarly, the estimates of the variance components from the fitted model suggested that study explained most of the shared variability among observations (study *σ*^2^ = 0.58, site-year *σ*^2^ = 0.04). The effects of seed-applied neonicotinoids on natural enemies compared to pyrethroid controls appeared to be homogenous (*Q* = 369.6, df = 383, *P* = 0.68), with an *I*^2^ indicating that 100% of the variation in effect sizes could be explained by random sampling error.

When Ohnesorg, Johnson & O’Neal (2009) was excluded from the dataset, the confidence intervals were smaller but the conclusion remained the same: seed-applied neonicotinoids and pyrethroids had similar influences on natural-enemy populations (Fig. 3). The overall mean effect size was very close to zero, −0.02 or 0.03, for models that did or did not include random effects of site-year nested in study. The variance components for study and site-year were small in this model (study *σ*^2^ = 0.01, site-year *σ*^2^ = 0.0008). Consistent with the first analysis, there was no evidence of heterogeneity in these effects (*Q* = 316.9, df = 375, *P* = 0.99, *I*^2^ = 0).

Again, we proceeded with the ‘moderator’ model to test whether various factors influenced the magnitude of effect size. In this case, the omnibus test suggested that the moderators did not explain significant variation in effect size (all studies: *Q*_M_ = 4.50, df = 6, *P* = 0.61; excluding (Ohnesorg, Johnson & O’Neal, 2009): *Q*_M_ = 3.40, df = 6, *P* = 0.76). This result is not surprising given the zero estimate of heterogeneity in this dataset, and suggests that the effect size of seed-applied neonicotinoids compared to pyrethroids is fairly consistent across the dataset, except for the observations associated with one outlying study.

### Statistical assumptions, potential biases, and robustness of results

We found little evidence of collinearity among our moderators. Pairwise correlations among moderators were centered near zero and mostly small (No-insecticide control: 84% < 0.2, median: −0.03, mean: −0.003, range: −0.56 to 0.52; Pyrethroid control: 80% < 0.2, median: −0.05, mean: −0.05, range: −0.51 to 0.34). Generalized variance inflation factors for moderators in both analyses were all less than two, again suggesting that collinearity among our predictors was minimal (Kutner et al., 2005).

For each of the datasets, diagnostic plots identified a handful of outliers with large standardized residuals (absolute value > 3). However, these outliers had little leverage, and removing them did not appreciably change parameter estimates or the outcome of significance tests (data not shown; results can be replicated using Codes S1 and S2).

We found no evidence of publication bias in our datasets. The distributions of effect sizes were bell-shaped with no evidence of an absence of observations near zero (Figs. S1 and S2). Furthermore, publication status was not a significant moderator of effect size in the no-insecticide comparison (Table 3). Rosenberg’s fail-safe N suggested that over 10,000 null observations would be necessary to render non-significant the difference between seed-applied neonicotinoids and insecticide-free controls. The ‘trimfill’ analysis estimated zero missing observations for each of the datasets, lending further support to the absence of publication bias.

The ‘leave one out’ analyses showed that our results for the no-insecticide comparison were fairly robust to the exclusion of particular studies. The estimated intercepts, slopes, and confidence intervals were quite similar across the analyses, and the overall effect of neonicotinoids on natural enemies was consistently negative (data not shown; results can be replicated using Code S1). In two out of twenty cases (Ohnesorg, Johnson & O’Neal, 2009; Sotelo-Cardona, 2010), leaving a study’s observations out of the analysis changed the omnibus test of moderators from significant to non-significant. This is perhaps not surprising given that the heterogeneity in this dataset was generally low. Overall, the sensitivity analysis suggested that no particular study was overly influential in the finding of a negative effect of seed-applied neonicotinoids on natural enemies compared to insecticide-free controls, but that the difference in this effect between insects and other arthropods should be tested in future studies.

As discussed previously, for the effect of seed-applied neonicotinoids versus pyrethroids, the ‘leave one out’ analysis revealed that one study (Ohnesorg, Johnson & O’Neal, 2009) had a fairly large influence on the width of confidence intervals. Nonetheless, we reemphasize that regardless of the inclusion of this study or the model used to estimate effect sizes, the confidence interval for this comparison always enclosed zero, suggesting little to no difference in the influence of seed-applied neonicotinoids and pyrethroids on natural-enemy abundance. Excluding one study that used seed-applied pyrethroids as the comparison group (Baker et al., 2002) did not change our results. It is notable that there were fewer studies available that investigated pyrethroid insecticides versus no-insecticide controls (8 studies versus 20 studies), and very few studies investigating other insecticide classes (Table S1), a discrepancy that could be addressed in future research.

### Effect of seed-applied neonicotinoids on predator–prey ratios in soybeans

Seven soybean studies reported sufficient information to calculate predator–prey ratios. The focal prey in five of the studies was the soybean aphid (*Aphis glycines*), while a sixth study focused on herbivorous thrips and a seventh focused on pest slugs (mainly *Deroceras* spp.). Aphids and thrips are listed on the neonicotinoid label for soybeans and so could be considered ‘target pests,’ though in practice soybean aphids are often not controlled sufficiently with seed-applied neonicotinoids (Myers & Hill, 2014). Slugs are non-target pests because they are generally not susceptible to neonicotinoids (Douglas, Rohr & Tooker, 2015; Simms, Ester & Wilson, 2006).

For studies focusing on soybean aphids, plots planted with neonicotinoid-coated seeds had numerically lower predator–prey ratios than plots treated with foliar insecticides (neonicotinoids, pyrethroids, or pymetrozine) in 13 out of 16 comparisons (Fig. S3 ). In contrast, plots planted with neonicotinoid-coated seeds had numerically higher predator–prey ratios than untreated controls in 11 out of 16 comparisons. For the studies focusing on non-aphid prey (thrips or slugs), all three predator–prey ratios were numerically lower in neonicotinoid-treated plots than untreated controls (Fig. S3 ). These results suggest that seed-applied neonicotinoids have a stronger effect on aphids than on natural enemies, and that the tested foliar insecticides are even more selective. On the other hand, the limited data available for non-aphid pests suggest that seed-applied neonicotinoids reduce predator–prey ratios, which could signal a disruption of biological control. We caution that these conclusions are based on relatively few studies and pest/predator combinations, and lack an estimate of variability. Moreover, most of them are based on the ratio of a focal pest to the summed abundance of a relevant guild of generalist predators, and so do not take into account differences between natural enemy taxa in predation rates.

## Discussion

We performed a meta-analysis of field studies to determine the influence of seed-applied neonicotinoids on arthropod natural enemies of crop pests in North America and Europe. After gathering and synthesizing results from almost 1,000 observations gleaned from 20 studies, we found that seed-applied neonicotinoids: (1) reduced natural-enemy abundance and (2) reduced natural enemies similarly to foliar or soil-applied pyrethroids. Furthermore, the influence of seed-applied neonicotinoids on natural enemies differed by broad taxonomic group: insects were more strongly affected than non-insect arthropods such as spiders and mites. This last result suggests that reductions in natural-enemy populations associated with seed-applied neonicotinoids are at least partly a result of toxin exposure, rather than prey scarcity alone.

Seed-applied neonicotinoids reduced the abundance of natural enemies relative to no-insecticide controls, with an effect size (*d* =  − 0.30) corresponding to roughly 16% reduced abundance. This result was robust to different modeling choices and unexpectedly consistent across crop species and neonicotinoid active ingredients. For comparison, the mean effect of organic farming (versus conventional farming) on predatory insect abundance was estimated to be *d* = 0.49 (Bengtsson, Ahnström & Weibull, 2005). Both effect sizes suggest that synthetic insecticides can undermine natural-enemy populations, but the consequences of these reductions for ecosystem services are hard to predict given a lack of research relating predator abundance to biological control function and its economic value (Naranjo, Ellsworth & Frisvold, 2015). The one study in our dataset that explicitly related predator abundance to crop yield was our previous study in a no-till soybean system (Douglas, Rohr & Tooker, 2015). In that study, a 31% reduction in early season abundance of slug predators in neonicotinoid-treated plots corresponded to a 67% increase in slug abundance and an eventual 5% reduction in soybean yield. Incidentally, the season-long reduction in slug-predator abundance was 16%, very similar to the mean effect identified in this meta-analysis, suggesting that a reduction of this magnitude can have economic consequences. Future efforts to relate natural-enemy abundance to crop yield could also make use of the concept of ‘natural-enemy units,’ which help to consolidate diverse natural enemies into a single measure of pest-suppression potential (Bahlai et al., 2010; Hallett et al., 2014). Ultimately, it would be valuable to build the knowledge base necessary to fit models relating natural-enemy abundance to pest abundance and ultimately crop productivity, analogous to those recently developed for pollination services (Koh et al., 2016).

Our finding that seed-applied neonicotinoids can in some cases increase predator–prey ratios further highlights that natural-enemy abundance is not equivalent to biological control function. We stress that a formal analysis of predator–prey ratios was not possible, but generally seed-applied neonicotinoids tended to have a smaller effect on natural enemies than on pest aphids, and a relatively larger effect on natural enemies than on other pest taxa (slugs and thrips). This pattern is further supported by case studies in the literature. We are not aware of any systems in which seed-applied neonicotinoids have been associated with resurgence of target pests such as aphids; however, there are several examples where these seed treatments have been associated with increased abundance and sometimes economic outbreaks of non-target pests, including spider mites (Smith et al., 2013), slugs (Douglas, Rohr & Tooker, 2015), and late-season stem-boring caterpillars (Pons & Albajes, 2002).

Our finding that insects were more strongly affected by seed-applied neonicotinoids than were non-insect groups (mainly spiders and mites) suggests that toxin exposure is at least partly responsible for the overall negative effect we observed, and raises the question of how insect natural enemies are being exposed to these seed-applied toxins. Neonicotinoids can poison natural enemies through ingestion as well as contact with sprays or residues (Lucas et al., 2004; Torres & Ruberson, 2004; Wang et al., 2008). Possible exposure pathways include contact with soil or planting dust (Goulson, 2013), ingestion of contaminated prey (Douglas, Rohr & Tooker, 2015; Szczepaniec et al., 2011), and for some natural enemies, ingestion of pollen, nectar, or other plant products (Lundgren, 2009; Moser & Obrycki, 2009). The relative importance of the various exposure pathways in the field is unclear, but we did see a non-significant trend for soil-dwelling taxa to be more strongly affected than foliar-dwelling taxa. Typically ∼90% of seed-applied neonicotinoids remain in soil, rather than entering the growing crop plant (Goulson, 2013), and recent findings reveal a layer of elevated residues on the soil surface where many species are active (Limay-Rios et al., 2016). Soil exposures, therefore, appear to be an important area for future research, particularly because previous research has leaned toward foliar-dwelling taxa. Finally, although the pattern we observed is consistent with the toxin exposure hypothesis, we cannot rule out that different responses to seed-applied neonicotinoids by insects and non-insect taxa reflect differences in ecology rather than (or in addition to) toxin susceptibility. For instance, many spider species can endure very long periods of starvation (e.g., Anderson, 1974), and there are also important differences in mobility between insects and arachnids. The toxin exposure hypothesis could be tested more directly using semi-field enclosure studies that hold immigration and prey availability constant while changing the exposure of insects and other taxa to neonicotinoid residues.

In contrast to toxin exposure, there was insufficient evidence to conclude that prey scarcity contributed to reductions in natural-enemy abundance by seed-applied neonicotinoids, although this result may change with additional research. While functional group was not a significant moderator of natural-enemy response to seed-applied neonicotinoids, there was a trend in the direction we would expect if prey scarcity were involved (parasitoid >  predator > omnivore). The prey scarcity hypothesis is also supported by a case study on the multicolored Asian lady beetle, *Harmonia axyridis*. This species is an important predator of the soybean aphid, and its population dynamics in the American Midwest over the past two decades correlated with changes in abundance of its soybean aphid prey, which in turn correlated with use of seed-applied neonicotinoids (Bahlai et al., 2015). More generally, although seed-applied neonicotinoids do not always provide economic control of aphids, they do sometimes reduce their seasonal populations (Hallett et al., 2014; Heidel-Baker, 2012; Johnson et al., 2009; Ohnesorg, Johnson & O’Neal, 2009; Tinsley et al., 2012). In turn, aphids are key prey for many generalist predators in agricultural systems (Donaldson, Myers & Gratton, 2007; Symondson, Sunderland & Greenstone, 2002). Future research could test the relative importance of prey scarcity versus toxin exposure through field studies that manipulate prey density independently of neonicotinoid treatment.

We expected seed-applied neonicotinoids to reduce populations of natural enemies less than foliar or soil-applied pyrethroids, but aside from one outlying study (Ohnesorg, Johnson & O’Neal, 2009), this was not the case. The limited number of independent studies (eight) comparing neonicotinoids to pyrethroids may have affected this result, and we encourage more research in this area. That said, our finding is consistent with previous meta-analyses (Naranjo, 2009; Wolfenbarger et al., 2008) that found a negative effect of pyrethroids on predatory arthropods (versus transgenic *Bt* varieties) of similar magnitude to the negative effect we found for seed-applied neonicotinoids (versus untreated controls). Pyrethroids are the second most widely used class of insecticides in the world after neonicotinoids (Sparks, 2013), and are important alternatives to seed-applied neonicotinoids in North American and European field crops (Budge et al., 2015; Douglas & Tooker, 2015; Furlan & Kreutzweiser, 2015). Their use is therefore likely to increase if, when, and where neonicotinoid use is restricted. Foliar and some soil-applied pyrethroids have the advantage that they can be applied in response to economic pest populations, and can therefore be more compatible with integrated pest management than seed treatments, which are typically applied to the seed months before planting (Furlan & Kreutzweiser, 2015; Johnson et al., 2009). It is also worth noting that pyrethroids and neonicotinoids overlap in their acute toxicity to mammals (Tomizawa & Casida, 2005), although foliar or soil applications would likely also entail different exposures than seed applications. Additionally, in some cases seed-applied neonicotinoids may be replaced by cultural management tactics or nothing at all. While the full economic, human health, and environmental trade-offs of neonicotinoids versus pyrethroids and other pest management strategies are beyond the scope of this study, our results do suggest that seed-applied neonicotinoids are neither uniquely risky nor benign to an important group of non-target invertebrates.

Prior to our meta-analysis, the statistical results within and across studies in our dataset appeared highly variable, and a narrative review of these findings could characterize them as mixed. In fact, their measured effects were largely consistent with one another, as reflected in the low heterogeneity of effect sizes across our datasets. This apparent contradiction results from the modest size of the effect combined with the high variability of measurements in field studies, and emphasizes the importance of considering statistical power during ecological frisk assessment. Detecting a 20% reduction in natural-enemy abundance with 80% probability requires at least 15 plots per treatment for many predatory arthropod taxa (Prasifka et al., 2008), far more than most studies contributing to our dataset (typically three to six replicates per treatment). While increasing sample size is an obvious solution, logistical and funding constraints make this a challenge. We suggest that researchers interpret null results conservatively in light of statistical power. Periodic meta-analyses may be useful for drawing broader conclusions, as has been the case for transgenic *Bt* crops (Marvier et al., 2007; Naranjo, 2009). The datasets we compiled for this study and a dataset of neonicotinoid effects on bees (Lundin et al., 2015) together provide a foundation for ongoing meta-analyses on the influence of neonicotinoids on non-target species.

There are several important limitations of our meta-analysis that stem from constraints of the dataset we compiled. Because most studies measured natural-enemy abundance within a single field season, our results do not address the influence of seed-applied neonicotinoids on other important metrics like species diversity, sublethal effects on behavior, reproduction of long-lived species, or long-term effects on natural-enemy populations associated with chronic exposure. Furthermore, our dataset comprises manipulative plot studies that by their nature do not account for movement of natural enemies across landscapes. By focusing on seasonal mean abundance, we may have underestimated important but transient effects that occur only during the period soon after planting. Finally, our study was not able to address the influence of seed-applied neonicotinoids in cropping systems outside of North American and Europe. It is our hope that future meta-analyses will benefit from increased research in these areas.

## Conclusion

Using meta-analysis to synthesize the results from field studies in North American and Europe, we found that seed-applied neonicotinoids reduced natural-enemy populations similarly to foliar- or soil-applied pyrethroids. The negative effect of neonicotinoids on natural enemies was *d* =  − 0.30 ± 0.10 [95% CI], corresponding to a reduction of ∼16%. The patterns we observed suggest that seed-applied neonicotinoids exert their effects mainly on insect (versus arachnid) natural enemies, at least partly through toxin exposure. If restrictions on neonicotinoid use encourage substitution with pyrethroids, our results suggest that there will be little net effect on natural-enemy populations. In fact, the results of neonicotinoid restriction for natural enemies are likely to be complex, particularly because some pyrethroids can more easily be saved for those situations in which economically damaging pest populations occur. Finally, translating natural-enemy abundance into biological control function is not possible given current knowledge, and is an important area for future study.

##  Supplemental Information

10.7717/peerj.2776/supp-1 Data S1 Meta-analysis datasets This excel file contains datasets and associated metadata underlying a meta-analysis of the influence of seed-applied neonicotinoids on natural enemies of crop pests.

10.7717/peerj.2776/supp-2 Supplemental Information 2 Supplemental figures and tables **Table S1.** Studies included in our meta-analysis of neonicotinoid seed treatment effects on natural enemies (full references at end).
**Figure S1.** Weighted histogram for the effect of seed-applied neonicotinoids on natural enemies (relative to no-insecticide controls), color-coded by study (*n* = 607 observations from 56 site-years and 20 studies).
**Figure S2.** Weighted histogram for the effect of seed-applied neonicotinoids on natural enemies (relative to pyrethroid controls), color-coded by study (*n* = 384 observations from 15 site-years and 8 studies).
**Figure S3.** Change in predator-prey ratio (PP) as a result of seed-applied neonicotinoids, relative to control plots treated with either no insecticide or a foliar insecticide (calculated as 100% X (PP_Neonic_- PP_Control_∕*PP*_Control_)). Each point represents a treatment comparison within a given study; negative values indicate that predator-prey ratios were lower in the neonicotinoid-treated plots versus controls, while positive values indicate the opposite. Points with the same color were derived from the same study. PYR, pyrethroid; PYM, pymetrozine; IMI, imidacloprid; and THX, thiamethoxam.

10.7717/peerj.2776/supp-3 Code S1 Code for neonicotinoids vs. untreated controls Code used in the R statistical program to perform a meta-analysis estimating the influence of seed-applied neonicotinoids on natural enemies of crop pests, compared to controls treated with no insecticides.

10.7717/peerj.2776/supp-4 Code S2 Code for neonicotinoids vs. pyrethroid controls Code used in the R statistical program to perform a meta-analysis estimating the influence of seed-applied neonicotinoids on natural enemies of crop pests, compared to controls treated with soil- or foliar-applied pyrethroids.

10.7717/peerj.2776/supp-5 Supplemental Information 5 PRISMA checklist

## Additional Information and Declarations

### Data Availability

The following information was supplied regarding data availability: The raw data has been supplied as Data S1.
