# Supplementary material for: Meta-analysis reveals that seed-applied neonicotinoids and pyrethroids have similar negative effects on abundance of arthropod natural enemies
Source: PeerJ. 2016 Dec 7;4:e2776. doi: 10.7717/peerj.2776 (PMC5147019; doi:10.7717/peerj.2776)
Supplement: Supplemental Information 2 — Table S1. Studies included in our meta-analysis of neonicotinoid seed treatment effects on natural enemies (full references at end). Figure S1. Weighted histogram for the effect of seed-applied neonicotinoids on natural enemies (relative to no-insecticide controls), color-coded by study (n = 607 observations from 56 site-years and 20 studies). Figure S2. Weighted histogram for the effect of seed-applied neonicotinoids on natural enemies (relative to pyrethroid controls), color-coded by study (n = 384 observations from 15 site-years and 8 studies). Figure S3. Change in predator-prey ratio (PP) as a result of seed-applied neonicotinoids, relative to control plots treated with either no insecticide or a foliar insecticide (calculated as 100% X (PPNeonic- PPControl∕PPControl)). Each point represents a treatment comparison within a given study; negative values indicate that predator-prey ratios were lower in the neonicotinoid-treated plots versus controls, while positive values indicate the opposite. Points with the same color were derived from the same study. PYR, pyrethroid; PYM, pymetrozine; IMI, imidacloprid; and THX, thiamethoxam. [file peerj-04-2776-s002.pdf]

Supplemental Results

Table S1. Studies included in our meta-analysis of neonicotinoid seed treatment effects on natural enemies (full references at end).

Crop	Study abbrev.	Peer review	A.I.(s)	Alt. insect.	Functional group(s)	Habitat	Arth. class	Early samp. (%)	N plots	Plot size (m^2)
Barley	SoteloCardona2010		THX		M, Pr, Pa	F	A, I	25	16	112
Canola	Echegaray2009		IMI	OP, OC, PYR	O, Pr	S	I	20	3 - 4	100 - 752
Cotton/ Sorghum	Krauter2001		IMI		Pr	F	A, I	0	3	110000
Maize	Ahmad20052006	X	CLO	PYR	M, O, Pr	F, S	A, I	0 - 33	4	232
	Albajes2003	X	IMI		M, Pr	F, S	A, I	20	4	7000
	AlDeeb2003	X	IMI, CLO	OP, PYR	M, O, Pr	F, S	A, I	0 - 33	4	23 - 28
	Babendreier2015	X	CLO	BIO, PYR	M, Pr	S	A, I	0	7 - 61	1
	Bhatti2005	X	IMI	Bt, PYR	M, O, Pr, Pa	F, S	A, Ch, I	20 - 33	4	335
	delaPoza2005 Farinos2008	X	IMI	Bt	M, Pr	F, S	A, Ch, I	0 - 20	3 - 4	1833 - 5500
	Harmon2006		CLO		M, O, Pr, Pa	F, S	A, Ch, I	66 - 100	4	729 - 1080
Soybean	Douglas2015	X	THX		M, O, Pr, Pa	S	A, I	25	6	1080
	Hallett2014	X	IMI, THX		Pr, Pa	F	A, I	20 - 25	3	111
	HeidelBaker2012 Carter2013		THX	PYR	Pr, Pa	F	I	0 - 10	4	1003
	Ohnesorg2009	X	IMI, THX	PYR, TRI	M, Pr, Pa	F	A, I	14	6	150
	Seagraves2012	X	IMI, THX			F	A, I	13	4	41
	Spigler2013		THX		Pr	F	I	65	4	409
	Tinsley2012	X	THX		Pr	F	I	0	4	70
Sugar beet	Baker2002		IMI, CLO, THX	PYR	M	S	A, I	33	4	108
Sunflower	Charlet et al. 2007	X	THX	CARB	Pa	F	I	0	4	24
Wheat	Schmidt et al. 2002		IMI		M	S	I	0	4	10000

Key to abbreviations: A.I. = active ingredient, Alt. insect. = alternative insecticide compared to neonicotinoid seed treatment, Early samp. (%) = the percentage of samples taken during the first 40 days of crop growth, IMI = imidacloprid, CLO = clothianidin, THX = thiamethoxam, BIO = biopesticide, Bt = transgenic Bt crop, CARB = carbamate, OC = organochlorines, OP = organophosphate, PYR = pyrethroid, M = mixed, O = omnivore, Pr = predator, Pa = parasitoid, F = foliar/aboveground, S = soil/belowground, I = insect, A = arachnid, Ch = chilopod

Supplemental Results

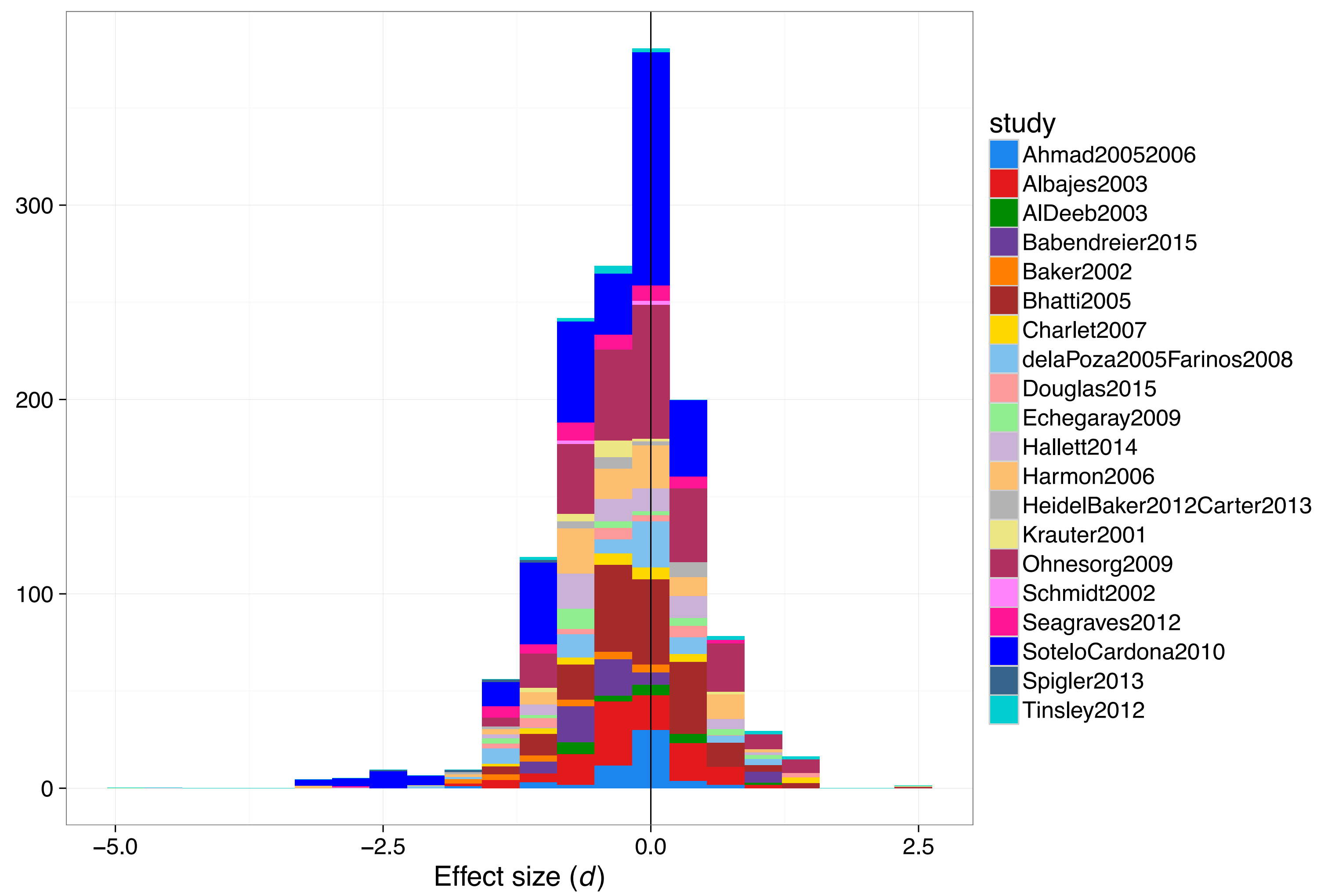

Figure S1. Weighted histogram for the effect of seed-applied neonicotinoids on natural enemies (relative to no-insecticide controls), color-coded by study (n = 607 observations from 56 site-years and 20 studies).

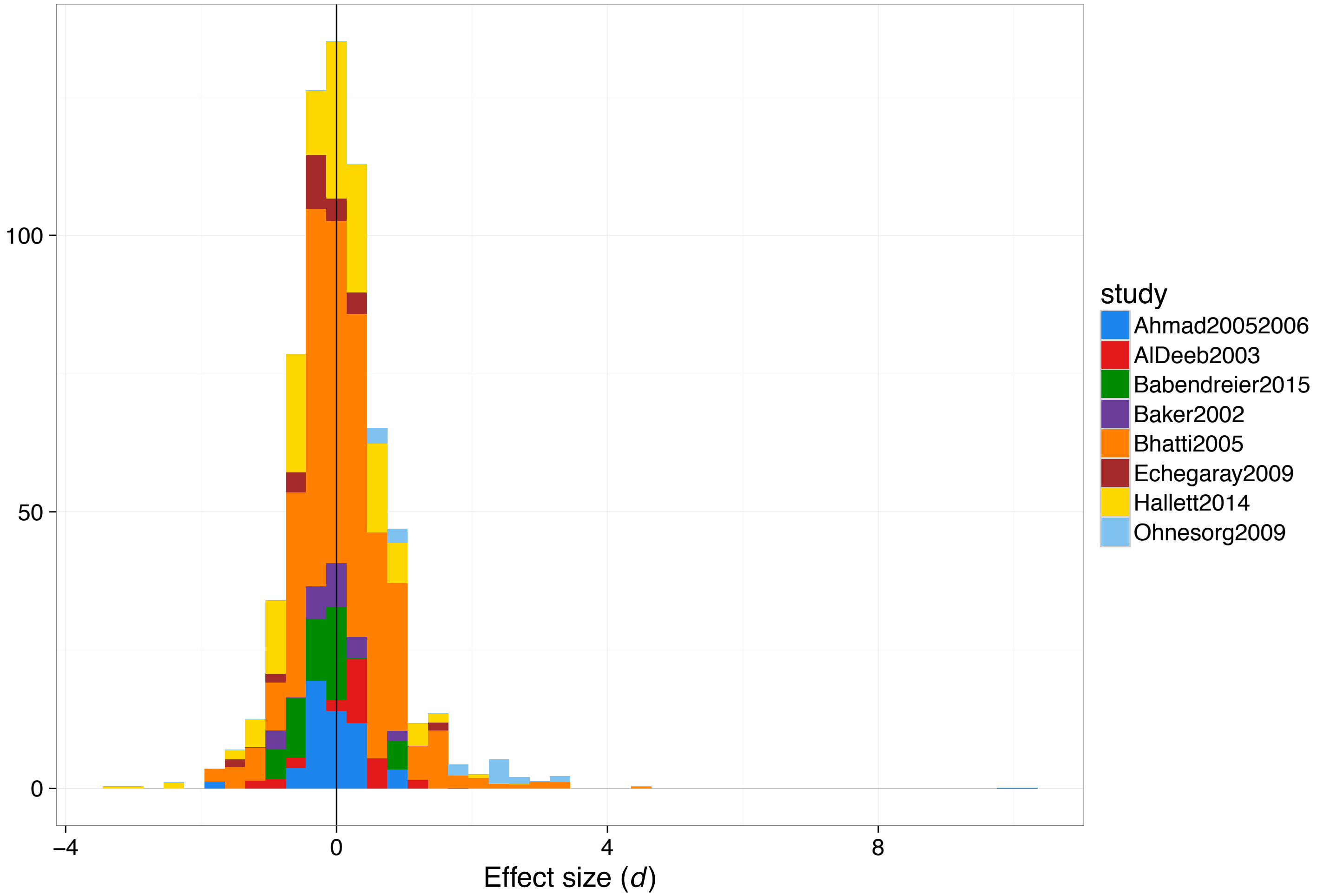

Figure S2. Weighted histogram for the effect of seed-applied neonicotinoids on natural enemies (relative to pyrethroid controls), color-coded by study (n = 384 observations from 15 site-years and 8 studies).

Supplemental Results

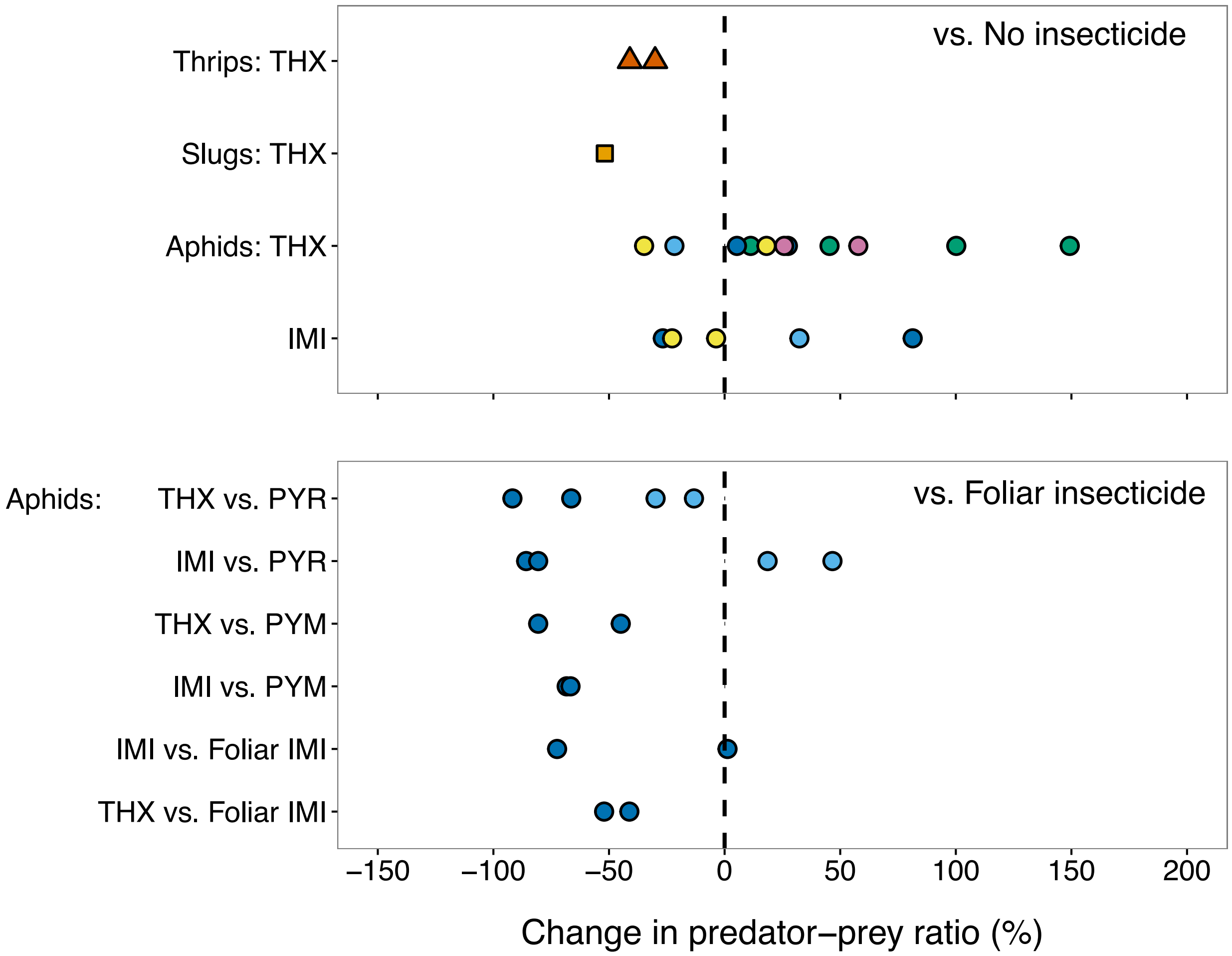

Figure S3. Change in predator-prey ratio (PP) as a result of seed-applied neonicotinoids, relative to control plots treated with either no insecticide or a foliar insecticide (calculated as 100% X (PP_{Neonic} - $PP_{Control)}$/$PP_{Control}$)). Each point represents a treatment comparison within a given study; negative values indicate that predator-prey ratios were lower in the neonicotinoid-treated plots versus controls, while positive values indicate the opposite. Points with the same color were derived from the same study. PYR = pyrethroid, PYM = pymetrozine, IMI = imidacloprid, and THX = thiamethoxam.

Supplemental Results

Supplemental References S1

Ahmad A, Wilde GE, Whitworth RJ, and Zolnerowich G. 2006. Effect of corn hybrids expressing the coleopteran-specific Cry3Bb1 protein for corn rootworm control on aboveground insect predators. *Journal of Economic Entomology* 99:1085-1095.

Ahmad A, Wilde GE, and Zhu KY. 2005. Detectability of coleopteran-specific Cry3Bb1 protein in soil and its effect on nontarget surface and below-ground arthropods. *Environmental Entomology* 34:385-394.

Al-Deeb MA, and Wilde GE. 2003. Effect of *Bt* corn expressing the Cry3Bb1 toxin for corn rootworm control on aboveground nontarget arthropods. *Environmental Entomology* 32:1164-1170.

Al-Deeb MA, Wilde GE, Blair JM, and Todd TC. 2003. Effect of *Bt* corn for corn rootworm control on nontarget soil microarthropods and nematodes. *Environmental Entomology* 32:859-865.

Albajes R, Lopez C, and Pons X. 2003. Predatory fauna in cornfields and response to imidacloprid seed treatment. *Journal of Economic Entomology* 96:1805-1813.

Babendreier D, Jeanneret P, Pilz C, and Toepfer S. 2015. Non-target effects of insecticides, entomopathogenic fungi and nematodes applied against western corn rootworm larvae in maize. *Journal of Applied Entomology* 139:457-467.

Baker P, Haylock LA, Gamer BH, Sands RJN, and Dewar AM. 2002. *The effects of insecticide seed treatments on beneficial invertebrates in sugar beet*. Farnham: British Crop Protection Council.

Bhatti MA, Duan J, Head G, Jiang CJ, McKee MJ, Nickson TE, Pilcher CL, and Pilcher CD. 2005a. Field evaluation of the impact of corn rootworm (Coleoptera: Chrysomelidae)-protected *Bt* corn on ground-dwelling invertebrates. *Environmental Entomology* 34:1325-1335.

Bhatti MA, Duan J, Head GP, Jiang CJ, McKee MJ, Nickson TE, Pilcher CL, and Pilcher CD. 2005b. Field evaluation of the impact of corn rootworm (Coleoptera : Chrysomelidae)-protected *Bt* corn on foliage-dwelling arthropods. *Environmental Entomology* 34:1336-1345.

Carter ME. 2013. Functional response and the effects of insecticidal seed treatment on the soybean aphid parasitoid, *Binodoxys communis.*M.S. Thesis. University of Minnesota.

Charlet LD, Aiken RM, Meyer RF, and Gebre-Amlak A. 2007. Impact of combining planting date and chemical control to reduce larval densities of stem-infesting pests of sunflower in the central plains. *Journal of Economic Entomology* 100:1248-1257.

de la Poza M, Pons X, Farinós GP, López C, Ortego F, Eizaguirre M, Castanera P, and Albajes R. 2005. Impact of farm-scale *Bt* maize on abundance of predatory arthropods in Spain. *Crop Protection* 24:677-684.

Douglas MR, Rohr JR, and Tooker JF. 2015. Neonicotinoid insecticide travels through a soil food chain, disrupting biological control of non-target pests and decreasing soya bean yield. *Journal of Applied Ecology* 52:250-260.

Echegaray FJ. 2009. Environmental effects of insecticides on non target predator and parasitoid insects. M.S. Thesis. Royal Roads University.

Farinós GP, de la Poza M, Hernández-Crespo P, Ortego F, and Castañera P. 2008. Diversity and seasonal phenology of aboveground arthropods in conventional and transgenic maize crops in Central Spain. *Biological Control* 44:362-371.

Hallett RH, Bahlai CA, Xue Y, and Schaafsma AW. 2014. Incorporating natural enemy units into a dynamic action threshold for the soybean aphid, *Aphis glycines* (Homoptera: Aphididae). *Pest Management Science* 70:879-888.

Harmon HE. 2006. Non-target effects of clothianidin seed-treated corn on the invertebrate community. M.S. Thesis. Towson University.

Heidel-Baker TT. 2012. Compatibility of soybean aphid integrated pest management strategies. Ph.D. Dissertation. University of Minnesota.

Krauter PC, Sansone CG, and Heinz KM. 2001. Assessment of Gaucho (R) seed treatment effects on beneficial insect abundance in sorghum. *Southwestern Entomologist* 26:143-146.

Ohnesorg WJ, Johnson KD, and O'Neal ME. 2009. Impact of reduced-risk insecticides on soybean aphid and associated natural enemies. *Journal of Economic Entomology* 102:1816-1826.

Schmidt T, Schmuck R, and Maus C. 2002. Data variability in carabid field studies (Coleoptera: Carabidae) and how to deal with small-scaled inhomogeneities of environmental conditions. *Bulletin OILB/SROP* 25:37-42.

Seagraves MP, and Lundgren JG. 2012. Effects of neonicotinoid seed treatments on soybean aphid and its natural enemies. *Journal of Pest Science* 85:125-132.

Sotelo-Cardona PA. 2010. Interactions among biological control, cultural control and barley resistance to the Russian wheat aphid, *Diuraphis noxia* (Kurdjumov), in Colorado, Kansas and Nebraska. Ph.D. Dissertation. Kansas State University.

Spigler MI. 2013. Effects of thiamethoxam seed treatments on nutritive sources available to *Orius insidiosus* say in Indiana soybean agroecosystems. M.S. Thesis. Purdue University.

Tinsley NA, Steffey KL, Estes RE, Heeren JR, Gray ME, and Diers BW. 2012. Field-level effects of preventative management tactics on soybean aphids (*Aphis glycines* Matsumura) and their predators. *Journal of Applied Entomology* 135:361-371.
